# Immuno-MALDI MS dataset for improved detection of HCVcoreAg in sera

**DOI:** 10.1016/j.dib.2019.104240

**Published:** 2019-07-08

**Authors:** Anna L. Kaysheva, Tatyana O. Pleshakova, Alexander A. Stepanov, Vadim S. Ziborov, Shanmuga S. Saravanabhavan, Balasubramanian Natesan, Alexander I. Archakov, Yurii D. Ivanov

**Affiliations:** a Institute of Biomedical Chemistry, Pogodinskaya St. 10, Moscow, 119121 Russia; b Department of Chemical Engineering, Anna University, Chennai, 600025, India

**Keywords:** HCVcoreAg, Aptamer, Atomic force microscopy, Mass spectrometry, Biospecific fishing, MALDI-MS

## Abstract

Complicated and large-scale challenge the contemporary biomedical community faces are development of highly-sensitive analytical methods for detection of protein markers associated with development of pathogenic mechanisms [2]. The atomic force microscopy (AFM) method in combination with specific fishing is unique among other analytical protein detection approaches; it allows visualization and counting of single protein molecules [3–6]. The present dataset focus on mass spectrometry method for detection of human hepatitis C virus core antigen (HCV core Ag) taking into account the potential modification with cations in blood serum samples, using mica chips for the atomic force microscopy (AFM-chips). To conduct specific protein fishing, we used flat AFM-chips preliminary sensibilized with molecular probes – aptamers, which are single-stranded DNA sequences. In our study we used four types of aptamers up to 85 nucleotides specific against the target protein – HCVcoreAg [3,4]. Working (n = 19) and control (n = 11) AFM-chips with aptamers were preliminarily immobilized on the surface in four zones and incubated in blood serum samples (See Supplementary fig. 1). Analysis of MS data regarding modification of marker protein peptides with Na+, K+, K2Cl+, and Na2Cl + ions enables to enhance the reliability of target proteins detection in the serum thereby demonstrating a high diagnostic potential.

**Table:** Specifications table

Subject area	Biology
More specific subject area	Biochemistry, immuno-MALDI MS analysis, protein detection
Type of data	Spectra, figures, tables
How data was acquired	Proteins identification was carried out using Autoflex III time-of-flight mass-spectrometer (Bruker, Germany), equipped with nitrogen laser with emission wavelength of 337 nm. To control efficiency of working AFM-chip surfaces enrichment, NTEGRA Prima atomic force microscope by NT-MDT (Zelenograd, Russia), AppNano cantilevers (USA), ACSTA model were employed.
Data format	Raw, filtered, analyzed
Experimental factors	To carry out specific enrichment of HCVcoreAg the AFM-chip with immobilized aptamers was incubated in blood serum samples. Subsequently, the AFM-chip was washed with deonized water and dried in presence of nitrogen flow. The trypsin digestion on the surface of AFM chip was used.
Experimental features	Mica with maximum surface elevation difference of up to 0.5 nm was used as AFM-chip. Four zones with immobilized aptamers were formed on the surfaces of working and control AFM-chips. Working AFM-chips were incubated in blood serum samples containing HCV RNA by PCR whereas, control AFM-chips were incubated in blood serum samples from healthy volunteers.
Data source location	Moscow, Russia
Data accessibility	The mass spectrometry data have been deposited to the web-site IBMC http://www.ibmc.msk.ru/content/lab_nanobiotech/MALDI_MS.zip
Related research article	Ivanov YD, Kaysheva AL, Frantsuzov PA, Pleshakova TO, Krohin NV, Izotov AA, Shumov ID, Uchaikin VF, Konev VA, Ziborov VS, Archakov AI. Detection of hepatitis C virus core protein in serum by atomic force microscopy combined with mass spectrometry. Int J Nanomedicine. https://doi.org/10.2147/IJN.S71776.

**Table:** 

**Value of the data** • MALDI MS method using aptamers as molecular probes allows to revelation of HCVcoreAg molecules from blood serum samples. • The use of specific aptamers against HCVcoreAg is preferable to the use of antibodies. • In the blood samples, cations interact with serum proteins including HCVcoreAg molecules. • Mass spectrometry identification of potential modified protein objects with K+ and Na + increases the quantity of the detected target peptides.

## 1 Data

**Fig. 1:**
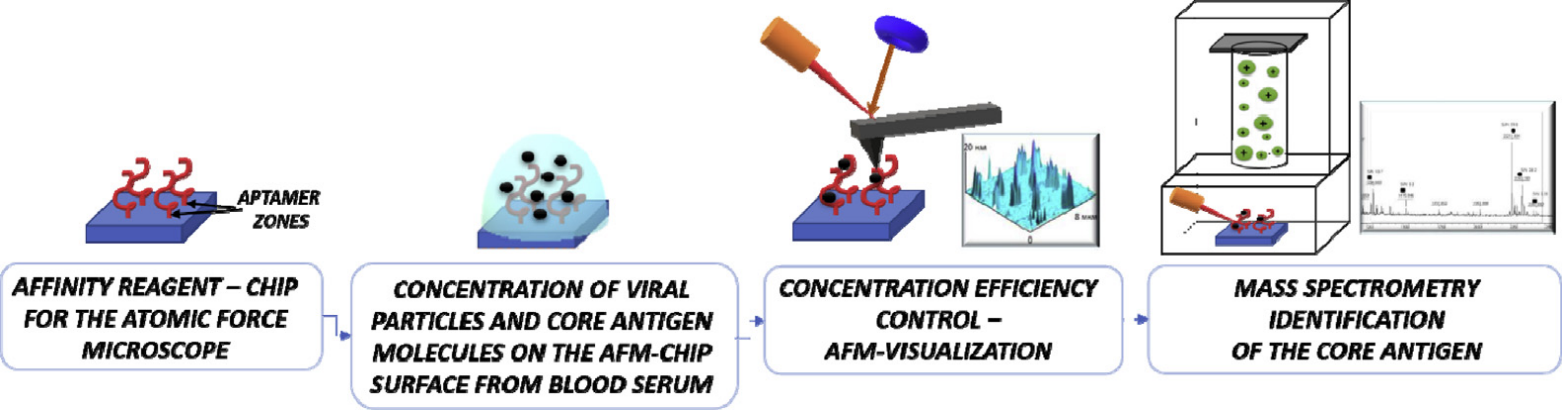
Pipeline for an AFM detection of target proteins specifically caught from the blood solution on the AFM-chip surface.

A sequence of procedures is presented in Fig. 1.

Early in our works we have showed, that the amount of nonspecifically sorbed protein on the surface is more than 2 times less than that obtained due to the affinity interaction of aptamer/antigen [2], [5].

**Fig. 2:**
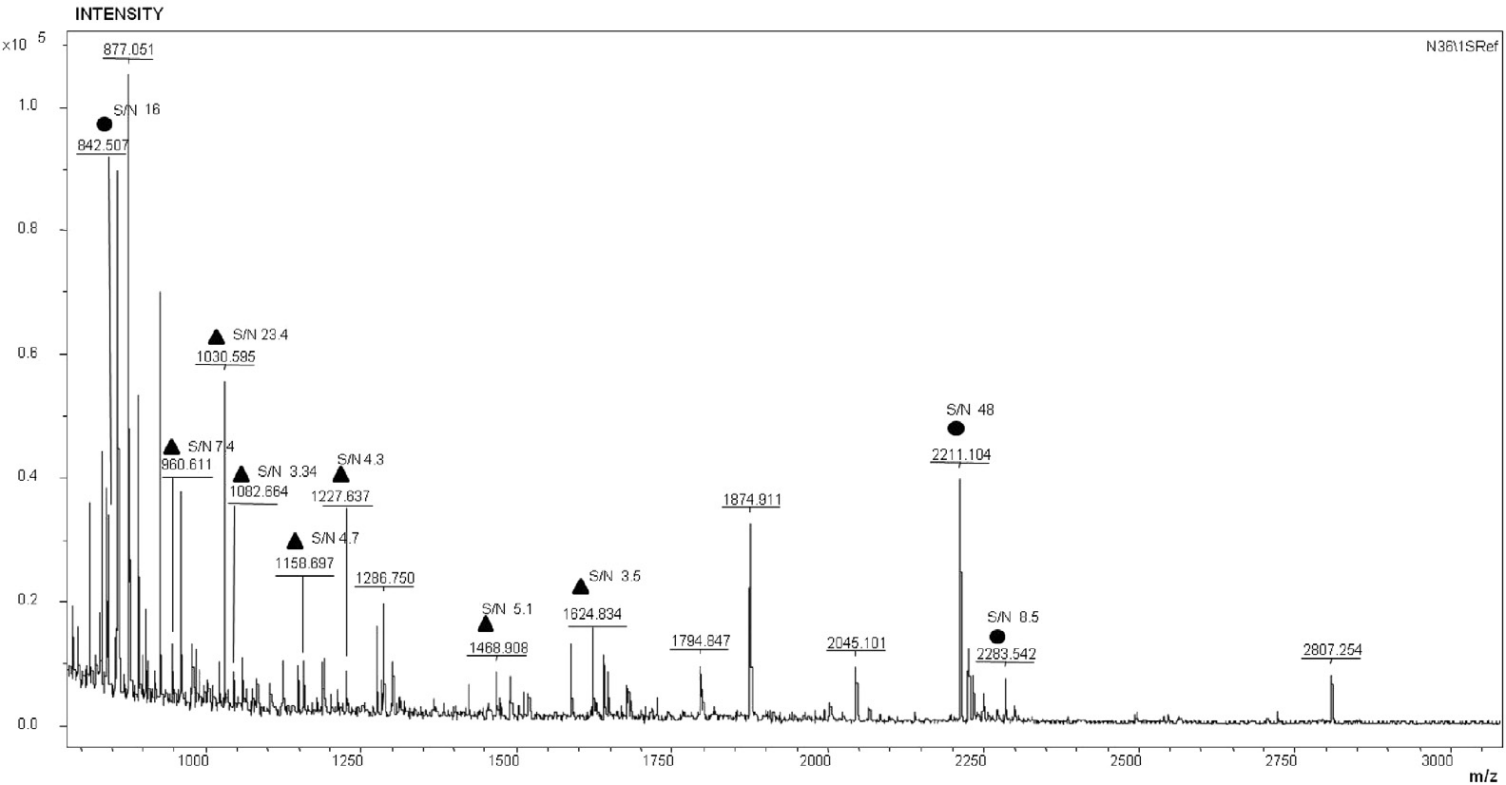
Standard mass spectrum of objects formed on the AFM-chip after incubation in the blood serum sample containing HCV RNA (by PCR). Black circles mark peaks of trypsin autolysis, black triangles – antigen peptides, S/N marks values corresponding to signal/noise ratio.

Standard MALDI MS spectrum obtained for a working AFM-chip after incubation in the blood serum sample containing HCV RNA (by PCR) is shown in Fig. 2.

The analysis of peptide maps taking into consideration the modifications of peptides HCVcoreAg with ions of K+, Na+ and their CL– ions, obtained for positive blood serum samples, demonstrated an increase in the number of identified peptides of the target protein 3 peptides on average (Table 1).

**Table 1:** Frequency of cation-modified peptides within HCVcoreAg in 19 blood serum samples positive for HCV RNA (by PCR).

Amino acid	Amino acid residues	m/z	Frequency peptide-cation			
			Na+	K+	Na_2_CL	K_2_CL
RGPRLGVR	40–47	910,5				2
ATRKTSER	48–55	948,5		4		
GRRQPIPK	60–67	951,5			2	
TSERSQPR	52–59	960,4		5		
RQPIPKAR	62–69	965,6	6			1
KTNRNTNR	42–47	1003,6			5	
MSTNPKPQK	41–47	1030,5	3			
GPRLGVRATR	41–50	1082,6	5			
KTSERSQPR	51–59	1088,6				1
MSTNPKPQKK	41–48	1158,6			1	
TSERSQPRGR	52–61	1173,6		3	4	18
GSRPSWGPTDPR	102–113	1312,7		1	1	
QPIPKARQPEGR	63–74	1376,7				5
GSRPSWGPTDPRR	102–114	1468,7	11			
MSTNPKPQKKTNR	41–51	1529,8	1			
GSRPSWGPTDPRRR	102–115	1624,8	11			
FPGGGQIVGGVYLLPR	24–39	1629,9			3	2
FPGGGQIVGGVYLLPRR	24–40	1786,0	6	2		6

**Fig. 3:**
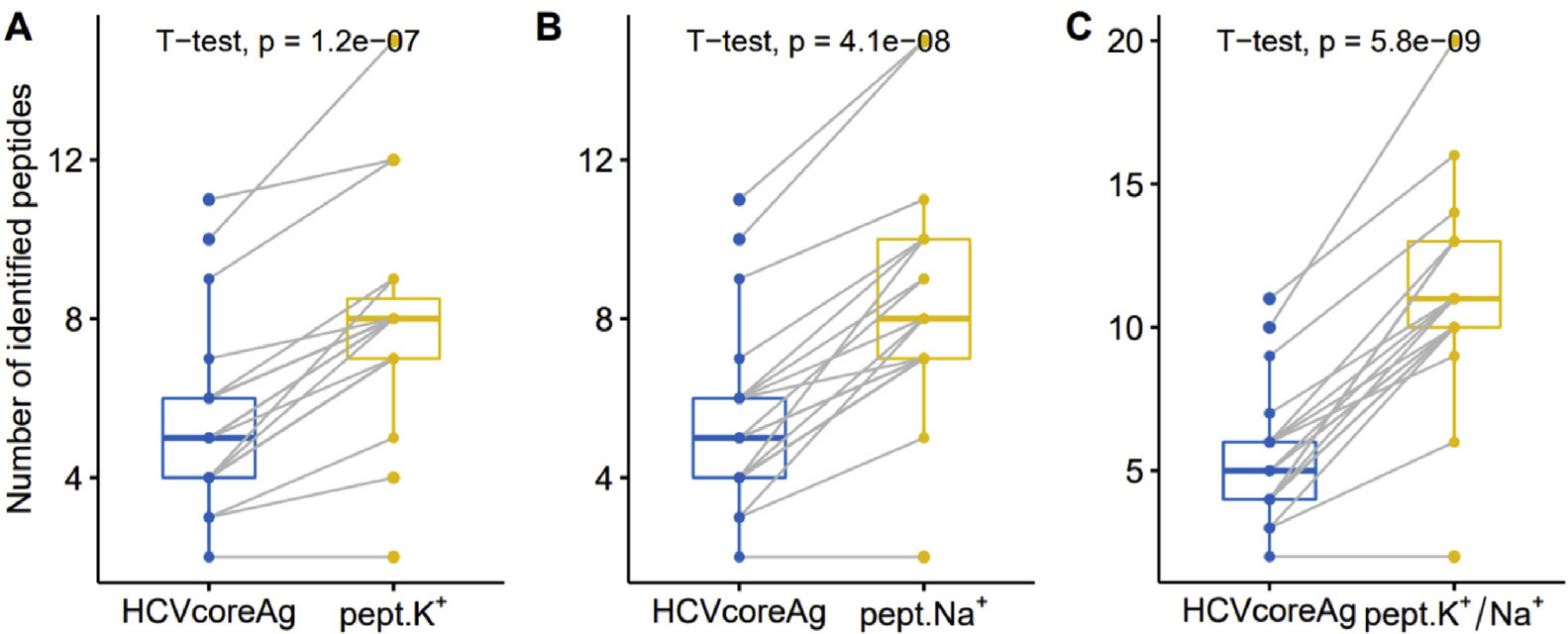
Tukey box plot of the increase in the number of identified peptides of HCVcoreAg due to peptides that capture potassium (A), sodium (B), and both (C). All p-values are from paired T-test for a difference in means.

The MALDI MS analysis of protein composition on AFM-chip surface with immobilized aptamers after incubation in 19 samples of blood serum samples positive for HCV RNA by PCR allowed for reliable detection of the target protein in 11 blood serum samples, whereas for 8 blood serum samples the detected number of peptide fragments of HCVcoreAg was insufficient for reliable identification. In MALDI MS false negative samples (the bar-zone in Fig. 3) were detected for up to 4 peptide fragments of the target proteins (See Supplementary Fig. 2). However, as it is clear from Fig. 3 the average of 3–5 cation-modified fragments of target protein were detected for most of samples.

## 2 Experimental design, materials and methods

*Reagents:* Dulbecco's phosphate buffered saline (DPBS, 10 mM*,* pH 7.4) (Pierce, USA); crosslinker 3,3′-dithiobis(sulfosuccinimidyl propionate) (DSP) (ТermoScientific, USA); (3-Aminopropyl)triethoxysilane (APTES), ammonium bicarbonate and dimethyl sulfoxide (DMSO) (Sigma-Aldrich, США); 1-Ethyl-3-(3-dimethylaminopropyl)carbodiimide (EDC), N-Hydroxysuccinimide (NHS), acetonitrile, isopropanol, formic acid (Merck, USA); trifluoroacetic acid (TFA), α-cyano-4-hydroxycinnamic acid (HCCA) (Sigma, USA); trypsin (Promega, USA); phosphate buffered saline concentrate with 0.05% tween-80 (Vector-Best, Russia). Solutions were made using the distilled water that were additionally treated with Milli-Q deionizer (Millipore, USA).

**Table:** *Aptamers* of four types against HCVcoreAg of at least 99% purity were purchased from Evrogen JSC (Russia). The sequences of NH_2_-(T) 10-Aptamer types are presented below.

Aptamer's name	Aptamer sequence
А12	5′–NH_2_–(T)_10_- ACGCTCGGATGCCACTACAGGCACGCCAGACCAGCCGTCCTCTCTTCATCCGAGCCTTCACCGAGCCTCATGGACGTGCTGGTGA-3′
А14	5′–NH_2_–(T)_10_- ACGCTCGGATGCCACTACAGTAACACACACAACTTAAAATCATACAAAAAAGAGTAAATGCCTCATGGACGTGCTGGTGA-3′
А15	5′–NH_2_–(T)_10_- ACGCTCGGATGCCACTACAGCCAAATACTACCGCAAAAACCACCTCCCCCTCGATAATAGCCTCATGGACGTGCTGGTGA-3′
А16	5′–NH_2_–(T)_10_- ACGCTCGGATGCCACTACAGTACCACACATGCAGACCCACACAAATACATACTAGAGACACCTCATGGACGTGCTGGTGA-3′

The thymine amino group and ten nucleotides were included into the nucleotide sequences of aptamers to improve its covalent immobilization efficiency on the surfaces of the AFM-chip, and to level up steric hindrances in interaction with target protein molecules [3].

*Serum samples* were kindly provided by the Department of Infectious Diseases in Children No. 1 of Russian National Research Medical University named after N.I. Pirogov of the Ministry of Health of Russia and G.N. Gabrichevsky Moscow Scientific Research Institute of Epidemiology and Microbiology of CPS of Russia. All serum samples were previously tested for the presence of HCV markers (HCV RNA, by PCR). Content of HCV RNA in patient serum presupposes intensive replication of virus in hepatocytes and hence, production and excretion of viral particles and HCV proteins in blood. From the PCR, all serum samples containing HCV RNA were considered to be HCV-positive. The patients gave their voluntary informed consent for participation in the study and use of their biomaterial.

### 2.1 Preparation of the AFM-Chip

Mica by SPI Inc. (USA), characterized by maximum surface elevation difference of up to 0.5 nm was used as AFM-chip. Four zones with immobilized aptamers (А12, А14, А15 and А16) were formed on the surfaces of working and control AFM-chips. Activation of the AFM-chip surfaces and immobilization of aptamers were carried out in accordance with the method detailed in article [3]. Arrangement of zones on the AFM-chip surfaces is presented in Supplementary Fig. 1. Working AFM-chips were incubated in blood serum samples containing HCV RNA by PCR whereas, control AFM-chips were incubated in blood serum samples from healthy volunteers.

To carry out specific enrichment of HCVcoreAg the AFM-chip with immobilized aptamers was incubated for 30 min in 1 mL of blood serum sample with vigorous mixing (850 r/min) in Thermomixer Comfort Shaker (“Eppendorf”, Germany) at 25 °C. Subsequently, the AFM-chip was washed thrice with deonized water for 30 minutes at 37 °C and dried in presence of nitrogen flow. Emulgen 913 solution at a concentration of 0.01% that has been used in our previous study [3], [4] was used as the wash buffer.

To control efficiency of working AFM-chip surfaces enrichment, NTEGRA Prima atomic force microscope by NT-MDT (Zelenograd, Russia), AppNano cantilevers (USA), ACSTA model were employed in the study. A detailed description of AFM-measurements and procedure for scanning process are presented in the article [3], [4].

### 2.2 Sample preparation of the AFM-Chip for mass spectrometry measurements

Working and control AFM-chips were cut along the contour of zones into four fragments corresponding to the zones of each aptamer and placed into individual marked Eppendorf's of 1.5 mL in volume. Trypsinolysis of protein objects on the surface of each zone of AFM-chip was carried out directly in marked Eppendorf's, into which 70 mcL of incubation solution including 1% acetonitrile, 0.1 μM of modified trypsin in 100 mM (рН 7.4) of bicarbonate buffer was added. Followed by the desalination of mixture with peptide fragments using Millipore ZipTip С18 (Sigma, USA) as per the manufacturer's protocol and supplied to the time-of-flight mass-spectrometer for measurements [6], [7].

### 2.3 Mass spectrometry measurements

Proteins identification was carried out using Autoflex III time-of-flight mass-spectrometer (Bruker, Germany), equipped with nitrogen laser with emission wavelength of 337 nm. Calibration of mass spectrometer was performed using the peptide calibration standard for positive ions in the reflector mode with voltage of 3.5–4.0 kV, the recorded mass spectrum was 750–3000 m/z with a pulse delay time of 200 ns. The peptide calibration standard was represented with following peptides (monoisotolic single-protonated ionic mass), bradykinin (757.3992 Da), angiotensin II (1046.5420 Da), angiotensin I (1296.6853 Da), R peptide (1347.7361 Da), bombesin (1619.8230 Da), renin (1758.9326 Da), ACTH fragment 1–17 (2093.0868 Da), ACTH fragment 18–39 (2465.1990 Da), and somatostatin (3147.4714 Da). The spectrum analysis excluded the peaks of trypsin matrix and autolysis. The mass spectrum was accumulated in auto mode until the sample spread on target was exhausted (usually 50,000 shots). To obtain mass spectra of the samples, the hydrolyzed mixture was mixed with excessive matrix (HCCA in 50% acetonitrile solution in 0.7% TFA) at the ratio ranging from 1:1000 to 1:10,000 and the resulting mixture was spread on MTP AnchorChip 384 target [6], [7], [8].

The mass spectra were processed with flexAnalysis 2.0 (Bruker, Germany). The protein identification was performed with Mascot proteomic search engine (http://www.matrixscience.com) using the protein sequencing data library SwissProt_2012. The following search options were selected: taxonomic group – hepatitis C virus, 2-missed hydrolysis sites, the acceptable measurement accuracy of monoisotopic masses was less than 100 ppm, methionine oxidation was indicated as a possible modification [5].
