# Supplementary material for: Immuno-MALDI MS dataset for improved detection of HCVcoreAg in sera
Source: Data Brief. 2019 Jul 8;25:104240. doi: 10.1016/j.dib.2019.104240 (PMC6656991; doi:10.1016/j.dib.2019.104240)
Supplement: Supplementary file 1 [file mmc1.doc]

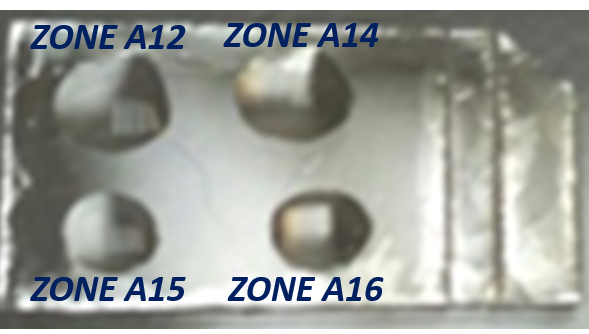


Figure 1 – AFM-chip with in cubation solutions of aptamers. Working zones (А12, А14, А15, А16) are marked in red, yellow, green, and blue correspondingly

**
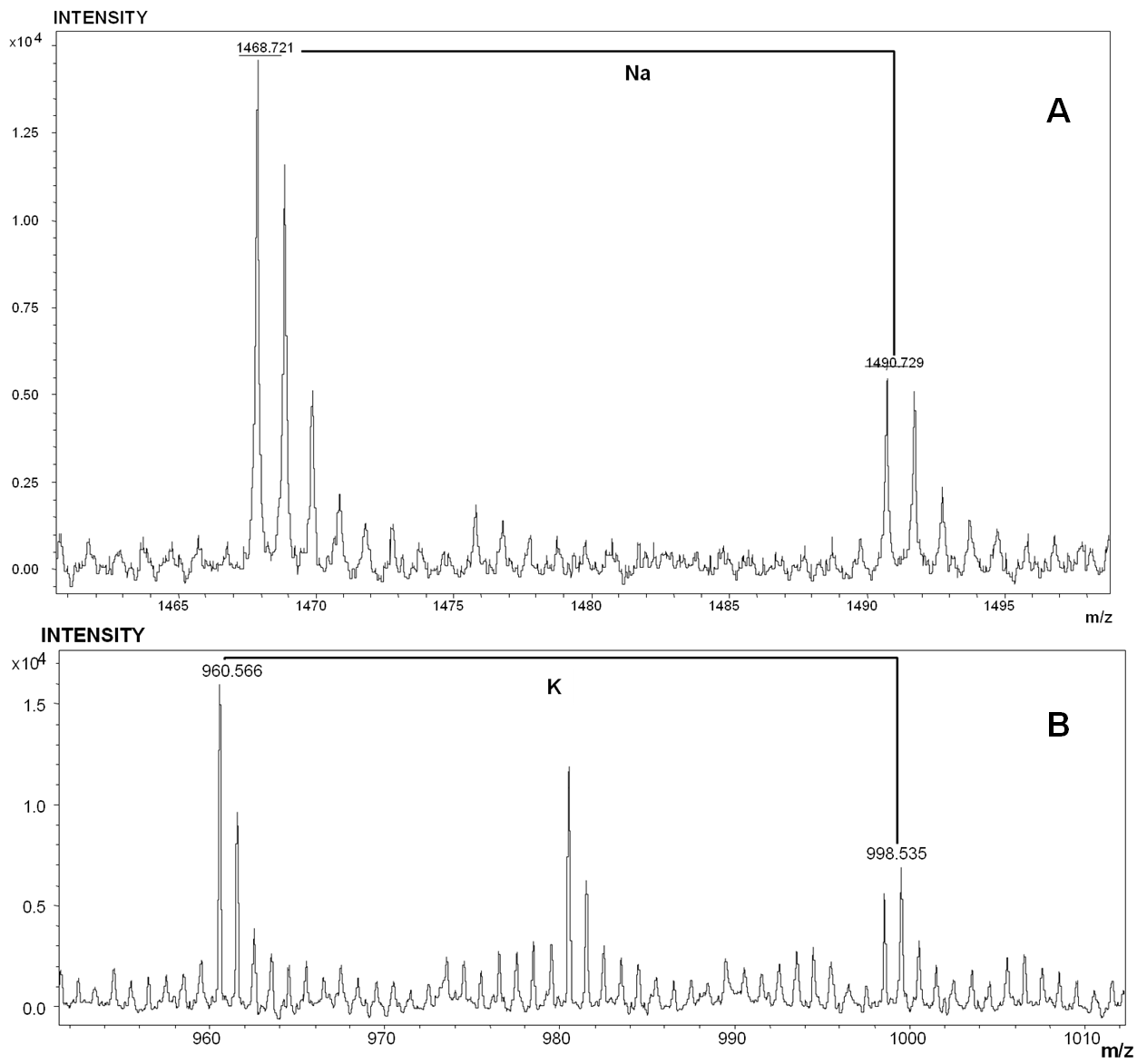
**

**
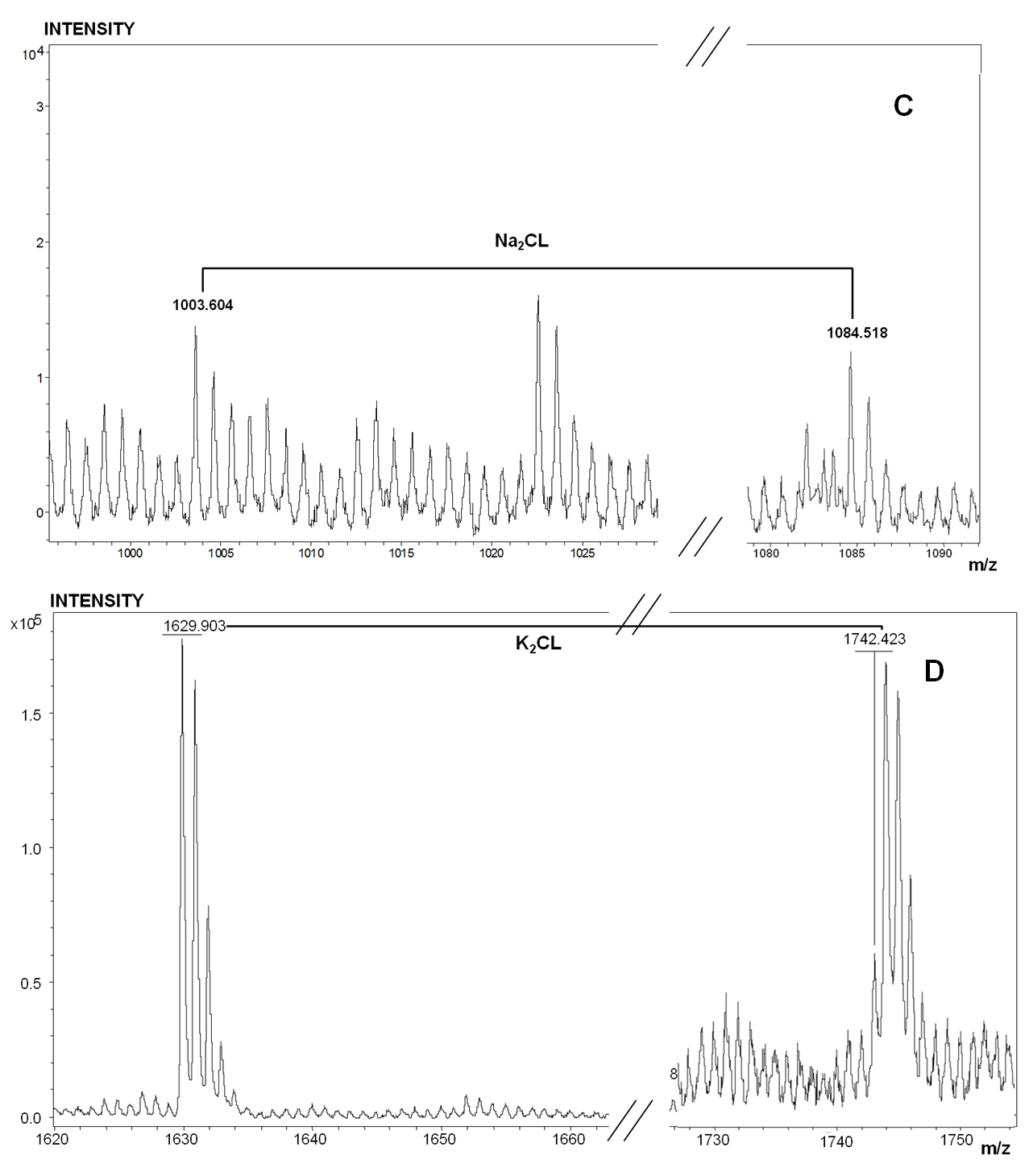
**

Figure 2 – Fragments of ionic mass spectra obtained for hydrolised objects from the surface of the working AFM-chip after incubation in the blood serum sample containing HCV RNA (by PCR). By their mass-charge characteristics (m/z) the ions correspond to peptides of HCVcoreAg and cation-modified variants. Peptide GSRPSWGPTDPRR (m/z 1468.7), modified with sodium cation (А), peptide TSERSQPR (m/z 960.5), modified with potassium (B), peptide KTNRNTNR (m/z 1003.6), modified with sodium chloride cation (Na_2_CL^+^) (C), peptide FPGGGQIVGGVYLLPR (m/z 1629.903) modified with potassium chloride (K_2_CL^+^) (D)
